# ELDER ABUSE PROSECUTION WITHOUT VICTIM COOPERATION: A DECISION-MAKING MODEL FOR PROSECUTORS

**DOI:** 10.1093/geroni/igae098.0141

**Published:** 2024-12-31

**Authors:** Catheryn Koss

**Affiliations:** California State University Sacramento, Sacramento, California, United States

## Abstract

Victims of elder abuse frequently refuse to cooperate with the prosecution of their abusers, especially if the accused is a spouse or other family member. What little guidance there is on how prosecutors should proceed in these situations tends to focus on evidentiary challenges and strategies to overcome them. While victims and prosecutors share the common overarching goal of addressing the problem of abuse, they may differ on how best to achieve that goal or at what cost. Victims may prioritize maintaining their relationships with their abusers and protecting their loved ones from the consequences of their actions. They may also fear loss of independence or placement in a long-term care facility. Prosecutors tend to prioritize preventing future abuse by keeping the abuser away from the victim and/or by reforming the abuser’s behavior. Prosecution sends a message that abuse is a serious crime that will not be tolerated, potentially having a deterrent effect by putting other potential abusers on notice that they too will likely be punished. This presentation will examine the common barriers to participation elder abuse victims face and the concerns elder abuse victims may have about prosecuting their abusers. Then, drawing on existing elder abuse scholarship as well as debates about this issue from the intimate partner violence literature, the presenter will share a novel decision-making framework - grounded in trauma-informed practices - that prosecutors and others may use to weigh the victim’s interests against the state’s interests in prosecuting the accused.

